# Efficacy of delayed treatment of China-made Peramivir with repeated intravenous injections in a mouse influenza model: from clinical experience to basal experiment

**DOI:** 10.1186/s12879-016-1589-9

**Published:** 2016-07-08

**Authors:** Zhengtu Li, Runfeng Li, Jing Li, Hui Xie, Yanbing Hao, Qiuling Du, Tingting Chen, Yimin Li, Rongchang Chen, Zifeng Yang, Nanshan Zhong

**Affiliations:** State Key Laboratory of Respiratory Disease, National Clinical Research Center for Respiratory Disease, Guangzhou Institute of Respiratory Disease, First Affiliated hospital of Guangzhou Medical University, (Guangzhou Medical University), Guangzhou, 510120 People’s Republic of China; Macau University of Science and Technology, Avenida Wai Long, Taipa, Macau, 519020 People’s Republic of China

**Keywords:** Peramivir, Delayed treatment, Survival rate, Lung index, Lung virus titer

## Abstract

**Background:**

China-made Peramivir, an anti-influenza neuraminidase inhibitor drug, is manufactured and widely used in China. Although effective if initiated within 48 h of the onset of symptoms, yet we observed that this drug shows an inconclusive efficacy if treatment is delayed in clinical. Thus we evaluated the efficacy of delayed treatment of China-made Peramivir in a mouse model.

**Methods:**

The mouse model of influenza infection was made and Peramivir was administered intravenously for 5 days following infection, and weight loss, lung index, viral shedding and survival rates were monitored.

**Results:**

Peramivir (60 mg/kg · d, repeated intravenous injections, quaque die (QD) × 5 days) enhanced survival rate and suppressed weight loss when treatment was initiated 24, 36, 48, or even 60 h post-infection (p.i.) (*p* < 0.01), compared with the virus-untreated group, and efficacy was abolished at 72 h p.i.. However the efficacy of delayed treatment was dose dependent, with the highest dose (90 mg/kg · d) even showing efficacy at 72 h p.i.. Furthermore, Peramivir (60 mg/kg · d, repeated intravenous injections, QD × 5 days) also reduced the lung virus titer 24 and 36 h p.i. on day 5, and even at 48 and 60 h p.i. on day 7 after infection, and the lung index was also improved. What is interesting that the concentration of the drug was maintained in blood after infected.

**Conclusions:**

Delayed treatment with China-made Peramivir can reduce the severity of influenza disease, accelerate viral clearance and enhance the survival rate. This drug therefore shows good efficacy and is a promising candidate to control the influenza epidemic in China.

## Background

Influenza virus poses a significant threat to human health, leading to considerable morbidity and mortality worldwide. Influenza pandemics, such as that caused by the influenza A H1N1pdm2009 virus that began in Mexico in early 2009 and spread globally [1–3], result in significant rates of hospitalization and death [4, 5]. Epidemic spread within a population can also lead to severe illness and death [6]. High rates of influenza infection are reported in China, particularly in the south of the country. The Centers for Disease Control (CDC) of China have reported data indicating two seasonal peaks in the number of cases of influenza infection in southern China each year, including human infections with highly pathogenic avian influenza viruses, such as H7N9/H10N8/H5N6 [7–9]. Therefore, more anti-viral drugs are needed in China to help combat this serious human infection.

The anti-influenza neuraminidase inhibitor drugs (for example, Oseltamivir, Zanamivir and Peramivir) are commonly used to prevent and treat influenza infection [10]. The M2 ion channel blockers (for example, amantadine and rimantadine) are less effective because of high drug resistance and adverse side effects [11–14]. Of these neuraminidase inhibitors, only Oseltamivir and Peramivir are manufactured in China and therefore most widely used in this country. Previous clinical trials indicated that treatment with neuraminidase inhibitors needed to be initiated within 48 h of the onset of symptoms [15–19]; however, owing to the delayed diagnosis of pathogens, anti-viral treatment in China was usually initiated after this time period, for example H7N9 patients were treated with neuraminidase inhibitors beyond 48 h after the onset of illness [20]. Furthermore, China-made Peramivir was also used to treat H7N9 patients in Guangzhou who had failed to respond to Oseltamivir, but this drug showed variable delayed treatment efficacy [21]. Therefore, research is required into the efficacy of delayed treatment with neuraminidase inhibitors, particularly China-made Peramivir, which is approved as an intravenous neuraminidase inhibitor [22] and showed a degree of efficacy clinically in patients in Guangzhou [21].

To investigate the efficacy of delayed treatment of Peramivir, manufactured in China, we employed a mouse model of influenza infection. Peramivir was administered intravenously, started at 24, 36, 48, 60 or 72 h p.i., for 5 days following infection, and viral shedding and survival rates were monitored to assess the delayed efficacy of Peramivir.

## Methods

### Viruses

Viruses used in this study included Influenza A H1N1pdm2009 (A/GZ/GIRD07/09, H1N1), isolated from fever patients in Guangzhou city between June and October 2009 and identified by genome sequencing, and Influenza B (B/Guangzhou/GIRD08/09), purchased from the CDC. These viruses were bred in the allantoic cavity of 9-d-old embryonated chicken eggs for 48 h at 35 °C, then 12 h at 4 °C, following which the harvested viruses were preserved at −80 °C prior to use. From these viruses, mouse lung-adapted variants were generated by repeated infection of mice. The 50 % lethal dose (LD_50_) of mouse, a simple method of estimating 50 % endpoints, was determined.

### Animal experiment design

BALB/C mice (6–8 weeks old) were purchased from Guangdong Medical Laboratory Animal Center (Guangzhou, China). The mice were housed in specific-pathogen-free facilities, bred, and genotyped according to the vendor’s protocols.

For lung index and lung virus titer determination, mice were treated with saline (50 μL) or virus (H1N1pdm2009: 3 LD_50_; influenza B: 10 LD_50_; in 50 μL saline) by intranasal inhalation with ether anesthesia. The experimental group were treated with Oseltamivir (purchased from EP company, lot 005171; dissolved in saline) via the oral route, or Peramivir (a gift from Hunan Nonferrous Kay Platinum Biological Pharmaceutical Co., LTD, China; lot 20140806; dissolved in saline) via tail intravenous injection. The normal control group and virus control group mice were treated with saline via oral administration. The drugs were administered for 5 days starting at 24, 36, 48, 60 or 72 h after infection, and mice were euthanized 5 or 7 d after influenza virus infection.

For the death protection experiments, mice were challenged with viruses, treated with Oseltamivir or Peramivir for 5 days, then observed over 15 days. Mice that died during drug treatment were treated as abnormal deaths. Finally, survival rates and average survival times were calculated.

For pharmacokinetic evaluation, mice were infected with H1N1pdm2009 virus, then the normal control group and the virus group were treated with Peramivir (60 mg/kg) after 48 h infection. Blood was collected from the suborbital vein at 0.5, 5, 10, 15, 20, 30, 40, 60 and 90 min after treatment. About 500 μL of blood per sample was collected into heparinized tubes and then immediately centrifuged at 4000 × *g* for 10 min. The plasma obtained was stored at −20C until analysis.

### Lung index determination and lung virus titer determination

Mice were euthanized at 5 or 7 d after virus challenge. Lung tissues were harvested and weighed. The lung index was expressed as the ratio of mean lung weight to mean body weight (lung index = lung weight/body weight × 100). The lung samples were homogenized in 1 mL of PBS, centrifuged at 10,000 *g*/min for 10 min, then the supernatant was collected. Virus titer was determined by the cytopathic effect method [23].

### Drug pharmacokinetic determination

#### Standard and sample preparation

Primary working solution of Peramivir was prepared by dissolving an accurately weighed amount (to 0.01 mg precision) in acetonitrile: water (1:1) to yield 1 mg/mL. For the assay of Peramivir in plasma samples, working standard solutions at 0.1, 0.25, 2.5, 5, 10, 20, 30, 60 and 20 μg/mL concentrations of Peramivir in acetonitrile: water (1:1) were prepared. Calibration standards of Peramivir were prepared by spiking 100 μL of fresh blank lean mice plasma with 10 μL of a standard Peramivir working solution, producing calibration samples with plasma concentrations of 0, 50, 100, 500, 1000, 2000, 4000, 6000, 12000 and 40000 ng/mL. The internal standard (IS) stock solution was prepared at 1 mg/mL in acetonitrile: water (1:1) in 1 mg/mL and was further diluted in acetonitrile to yield a working standard solution of 400 ng/mL. All solutions were stored at 4 °C and brought to room temperature before use.

To 100 μL of sample or blank plasma, a 10-μL aliquot of the IS solution (final concentration, 400 ng/mL), 10 μL of acetonitrile: water (1:1) or standard working solution was added. The mixture was extracted with 800 μL of ethyl acetate by shaking for 15 min. After centrifugation at 14,000 × *g* for 10 min, the upper organic layer was separated and evaporated at 45 °C under a stream of nitrogen in a Turbo Vap evaporator (Zymark, Hopkinton, MA, USA). The residue was reconstituted in 100 μL of the mobile phase, and then mixed by vortex ING. A 20-μL aliquot of the resulting solution was injected into the LC-MS/MS system for analysis.

#### HPLC-MS/MS conditions

The LC-ESI-MS/MS system consisted of a nanospace HPLC system (NANOSPACE 1312, Japan) coupled to a Q Trap™ 4000 hybrid triple quadruple linear ion trap mass spectrometer (Applied Biosystems/MDS Sciex, Concord, Ontario, Canada). Data were processed using the Analyst™ 1.5 software package (Applied Biosystems, MA, USA). Chromatographic separation was performed on a Luna C18 column (2.1 × 50 mm, 1.7 μm; CA, USA) at ambient temperature. The flow rate was 0.4 mL/min and the injection volume was 10 μL.

For LC-ESI-MS/MS detection, mobile phase A comprised water with 0.1 % formic acid and phase B comprised acetonitrile with 0.1 % formic acid. In the LC gradient profile, mobile phase B comprised 10 % (v/v) at the start, increased linearly to 100 % from 0.5 to 2.2 min and then returned to 10 % at 3.2 min, with a total run time of 4.0 min.

The mass spectrometer was operated using an ESI source in the positive ion detection mode for Peramivir and Oseltamivir (IS) determination. Acquisition was performed in the MRM mode using m/z values of 329.2/270.3 and 313.1/208.2 for Peramivir and IS, respectively. The optimized instrument parameters for monitoring the analytes by MS were as follows: source temperature, 650 °C; curtain gas, 30 psi; nebulizing gas, 80 psi; turbo ion spray gas, 70 psi; collision gas, medium; dwell time 100 ms.

### Statistical analysis

Data are presented as the mean ± SD. All data were analyzed using GraphPad and SPSS 17.0 software. Weight loss data were checked by repeated measurements and a mixed model multivariate analysis of variance. Statistics were analyzed with ANOVA or the two-tailed Student’s *t*-test. The probability of mouse survival was estimated by the Kaplan-Meier method and further analyzed by log-rank pairwise tests over strata. The pharmacokinetic profile and parameters of Peramivir were analyzed using DAS 2.0 pharmacokinetic software. Statistical significance was set as *p* < 0.05; *p* < 0.01 and *p* < 0.001 were considered as more significant differences.

## Results

### Clinical anti-viral treatment features of H7N9 patients in Guangdong province, China

In previous clinical trials, neuraminidase inhibitors only showed anti-influenza efficacy if administered within 48 h of the onset of symptoms [15–19]. However, we found a good treatment efficacy of neuraminidase inhibitors beyond 48 h, even up to 22 days after the onset of symptoms (Table 1). In our observations, H7N9 patients were usually treated late with anti-viral drugs, with the earliest treatment time being 7 days after the onset of illness among our patients (Table 1). Furthermore, Peramivir, manufactured in China, showed a degree of treatment efficacy (Table 1). The patient who died had a history of lung cancer.

**Table 1:** Clinical anti-viral treatment features of H7N9 patients in Guangdong province, China

Patient	Time (days) after onset of symptoms when anti-viral drugs were administered	Anti-viral drug used	Clinical outcome
1	22	Oseltamivir	Survival
2	8		
3	8		
4	7	Oseltamivir, China-made Peramivir	Survival
5	13	Oseltamivir, Zanamivir, China-made Peramivir	Death
6	9	Oseltamivir, Zanamivir	Survival
7	11		

*Some of these data were published by our research team, Zifeng Yang et al. [21]

### Delayed treatment with China-made Peramivir enhanced survival, suppressed weight loss and reduced lung virus titer

**Fig. 1:**
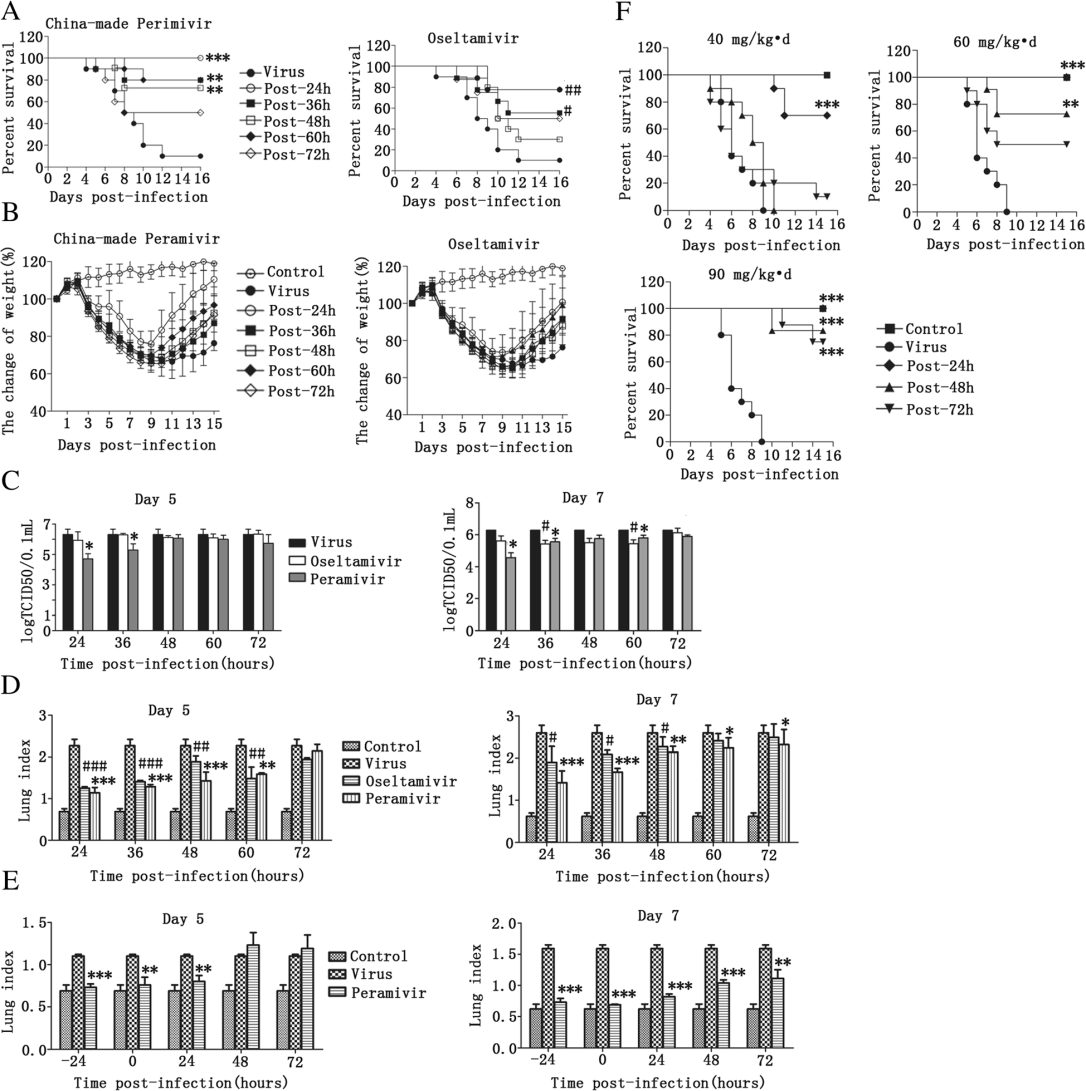
Delayed treatment of China-made Peramivir enhanced survival, suppressed weight loss and reduced lung virus titer. Mice were administered virus (H1N1pdm2009: 3 LD_50_; in 50 μL saline) via inhalation to induce acute lung inflammation, and were then treated with Peramivir (60 mg/kg · d, intravenous injection, QD × 5 days) and Oseltamivir (60 mg/kg · d, oral, QD × 5 days). Survival rate (**a**) and body weight (**b**) were determined daily for 15 d. Viral titers in the lungs (**c**) on days 5 and 7 were determined by the cytopathic effect method and the plaque assay. **d** Lung index of each group on days 5 and 7. The index was determined as the lung weight/final body weight (LW/BW). **e** Mice were administered virus (influenza B virus: 10 LD_50_; in 50 μL saline) via inhalation, and were then treated with Peramivir (40 mg/kg · d, intravenous injection, QD × 5 days). Lung index of each group on days 5 and 7 were calculated. **f** Mice were treated with different doses of Peramivir (40, 60 and 90 mg/kg · d), and the survival rate was determined daily for 15 d. Values are shown as mean ± SEM. *p < 0.05, **p < 0.01 and ***p < 0.001 Peramivir group versus virus control group; #*p* < 0.05, ##*p* < 0.01 and ###*p* < 0.001 Ositamivir group versus virus control group. Statistical analyses were performed consecutively with ANOVA or the two-tailed Student’s *t*-test

In the mouse influenza model, viral infection leads to high mortality and weight loss [24]. Therefore, the efficacy of delayed treatment of Peramivir was first evaluated with the survival rate, mean days to death and weight loss. The mouse model was set up as described in the Materials and Methods. Peramivir was administered at 60 mg/kg · d QD for 5 days and survival rates of 100, 80, 70 and 80 % were observed 24, 36, 48 and 60 h post-infection (p.i.), respectively (*p* < 0.01, compared with 10 % of the virus group), compared with 77.8, 55.6, 30 and 77.8 % survival rates in the Oseltamivir group under the same conditions (Fig. 1a). However, there was no efficacy for delayed treatment at 72 h p.i. for the two groups, and the mean number of days to death was only extended in the 24 h p.i. (>15 days) group compared with 8.33 ± 2.29 days in the virus group. Furthermore, the virus-infected mice exhibited a tendency toward weight loss from day 3 p.i., peaking on day 9. However, weight loss was suppressed in mice treated with Peramivir. At its peak (day 9), there was a clear difference (about 9 %) in weight loss suppression in the post-24-h treatment group only compared with the virus group. Meanwhile, on the last day of monitoring (day 15), there was significant weight loss suppression post-24 to post-72-h p.i. (*p* < 0.05, compared with the virus group). These effects were more marked in the Peramivir group compared with the Oseltamivir group (Fig. 1b).

The lung virus titer was also detected in each of the treatment groups. On day 5 p.i., the Peramivir treatment reduced lung virus titers only when treatment was initiated post 24 and 36 h (*p* < 0.05, compared with the virus group), which was an improved outcome compared with Oseltamivir treatment. However, on day 7, the lung virus titer was reduced by 24, 36 and 60 h (*p* < 0.05, compared with the virus group) for the Peramivir and Oseltamivir groups (Fig. 1c). Furthermore, the lung index showed an improvement at 24 to 72 h p.i. (*p* < 0.05, compared with the virus group) (Fig. 1d), indicating the increased treatment efficacy of Peramivir compared with Oseltamivir treatment. When Peramivir (40 mg/kg · d, QD for 5 days) was administered to influenza B virus-infected mice, the lung index was improved in the prevention and treatment model, even showing improved efficacy at 72 h p.i. on day 7 (*p* < 0.01, compared with the virus group) (Fig. 1e).

The efficacy of delayed treatment with Peramivir was dose dependent. At 40 mg/kg · d, the survival rate was only enhanced at 24 h p.i. (*p* < 0.05, compared with the virus group), and efficacy was observed at 48 h p.i. at 60 mg/kg · d (*p* < 0.05, compared with the virus group). Furthermore, the efficacy of delayed treatment of Peramivir was even observed at 72 h p.i. (*p* < 0.05, compared with the virus group) at 90 mg/kg · d (Fig. 1f).

### Concentration of China-made Peramivir can be maintained after virus infection in the mouse influenza A H1N1pdm2009 model

**Fig. 2:**
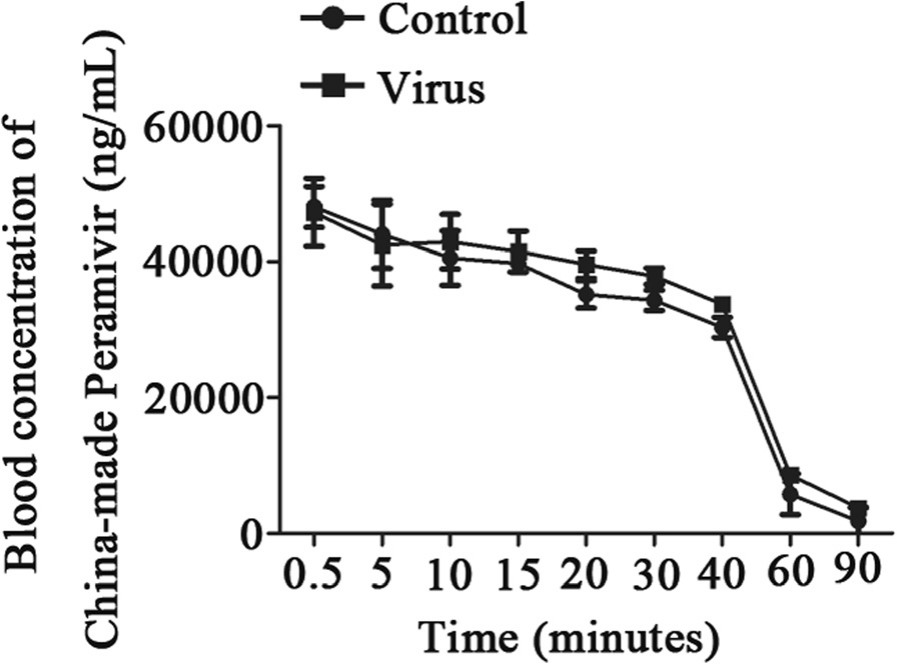
Concentration of China-made Peramivir in the blood of post-infected mice (concentration-time curve). Mice were administered virus (H1N1pdm2009: 3 LD_50_; in 50 μL saline) via inhalation, and were then treated with Peramivir (60 mg/kg · d, QD, tail intravenous injection). The blood was collected at different times, and the drug concentrations were detected. Values are shown as means ± SEM

A rough quantitation of blood Peramivir showed a lower concentration than that reported from a phase I clinical trial [25]. Furthermore, the previous study about the blood drug concentration of Peramivir was showed on the normal animal model [26]. We therefore investigated whether influenza virus infection affects the pharmacokinetics of Peramivir. As results shown, there were no significant differences in the concentration of Peramivir in the blood of influenza A H1N1pdm2009 virus-infected mice compared with normal mice, but slight increases were generally observed in the influenza A H1N1pdm2009 virus-infected mice (Fig. 2). Furthermore, the simulated pharmacokinetic parameters of Peramivir also showed no significant differences between influenza virus-infected mice and normal mice and; however, the mean retention time (MRT) was extended in the influenza virus-infected mice (Table 2).

**Table 2:** Pharmacokinetic parameters of China-made Peramivir

Parameters		Control mice	Virus-infected mice
AUC_(0-∞)_	μg/L/h	37307.80	38518.79
MRT_(0-∞)_	h	0.38	0.536
t_1/2_	h	0.289	0.341
CL	L/h/kg	1.662	1.61
V	L/kg	0.693	0.793
C_max_	μg	48037.54	47224.08

*AUC* area under curve, *MRT* mean retention time, *t*_*1/2*_ half a life, *CL* clearance, *V* volume of distribution, *C*_*max*_ maximum concentration

## Discussion

The present study provides evidence demonstrating that delayed treatment of China-made Peramivir can enhance the survival rate, inhibit virus replication in the lung, decrease the severity of viral lung lesions and remain at a stable concentration in the blood in the influenza virus infection mouse model.

Peramivir is a potent neuraminidase inhibitor of influenza that has been approved for intravenous use [27]. The in vitro activity of Peramivir has been shown to be comparable to, or better than, Oseltamivir carboxylate and Zanamivir [24]. Peramivir is unlike Oseltamivir (oral) and Zanamivir (inhalation) that are only suitable for treating cooperating patients, as it is suitable for the treatment of severely ill patients who cannot accept oral or inhaled treatments. For these reasons Peramivir was used to treat H7N9 patients (Table 1), but its clinical efficacy is yet to be confirmed. In this study, we observed that Peramivir treatment was usually delayed in patients owing to late diagnosis (Table 1). It was therefore important to determine the efficacy of delayed treatment of Peramivir, which we investigated in the mice model. Peramivir (60 mg/kg · d, intravenous injection, QD for 5 days) enhanced the survival rate of mice even when initiated 60 h p.i., compared with Oseltamivir (60 mg/kg · d, oral, QD for 5 days) (Fig. 1a). A previous report showed that a single intramuscular dose of Peramivir (10 mg/kg) administered at 24 or 48 h p.i. prevented death in a similar manner to Oseltamivir and Zanamivir treatment [24]. Another report showed that Peramivir, given as late as 60 h after infection, was able to prevent death in mice [28], similar to our findings, except that these studies administered a single intramuscular dose whereas we used repeated intravenous injections.

A clinical study found that repeated intravenous injections of Peramivir for 5 days conferred beneficial effects in influenza virus-infected patients at high risk of complications, and no major safety issues were identified [29]. In addition, another study reported no obvious signs of drug-related toxicity in mice after repeated intravenous injections of Peramivir at a dose of 40 mg/kg · d for 20 days [30]. They also found that repeated administration of Peramivir (40 mg/kg · d, intravenous injection, QD × 20 days) starting at 24, 48 or 72 h p.i. resulted in increased survival rates and reduced viral titers in the lungs. These findings demonstrated higher efficacy for delayed treatment than with Peramivir, potentially due to prolonged usage. In our study, we also found that the efficacy of delayed treatment of Peramivir was dose dependent (Fig. 1f). In the mouse model, Peramivir treatment showed no signs of toxicity or side effects at doses as high as 3000 mg/kg · d administered as a single dose or at doses of 1000 mg/kg · d administered for 5 days [31], indicating that prolonged treatment and higher doses of Peramivir may enhance the efficacy of delayed treatment. Clinically, we found that Peramivir even administered on day 7 p.i. also presented some treatment efficacy (Table 1), potentially indicating some differences between humans and mice and demonstrating the need for more clinical trials to evaluate the real efficacy of delayed treatment of Peramivir.

A single intramuscular injection of Peramivir (10 mg/kg) significantly reduces weight loss and mortality in mice infected with influenza A/H1N1, while Oseltamivir demonstrates no efficacy by the same treatment regimen. This has been suggested to be due to tight binding of Peramivir to the N1 neuraminidase enzymes [24]. In our study, we also observed the efficacy of delayed treatment of Peramivir combined with reduced lung virus titer (Fig. 1c/d), which may be potentially attributable to the tight binding of neuraminidase enzymes reducing virus replication. The drug concentration in blood is also important for drug efficacy. The lack of significant clinical effects may be due to the relatively low blood levels that were obtained following oral administration, indicating the low oral bioavailability of Peramivir (≤3 %). We demonstrated that the concentration of Peramivir in blood can be maintained after virus infection (Fig. 2), which may be another important factor in the efficacy of delayed treatment. However, this was only demonstrated in the animal model, so clinical trials will be needed to verify this in human subjects in the future.

## Conclusion

We have proven the efficacy of delayed treatment of China-made Peramivir at enhancing survival rates and reducing lung virus titers, and the maintenance of the drug concentration in blood following virus infection. These findings indicate the promising potential of China-made Peramivir for the treatment of influenza virus infections with delayed diagnosis, including avian influenza viruses, aiding control of influenza epidemics in China.

## Abbreviations

CDC, Centers for Disease Control; IS, internal standard; LD_50_, 50 % lethal dose; p.i., post-infection; QD, quaque die
